# Combining Simple Phenotyping and Photothermal Algorithm for the Prediction of Soybean Phenology: Application to a Range of Common Cultivars Grown in Europe

**DOI:** 10.3389/fpls.2019.01755

**Published:** 2020-01-29

**Authors:** Céline Schoving, Claudio Osvaldo Stöckle, Céline Colombet, Luc Champolivier, Philippe Debaeke, Pierre Maury

**Affiliations:** ^1^ Université de Toulouse, INRAE, UMR AGIR, Castanet-Tolosan, France; ^2^ Biological Systems Engineering, Washington State University, Pullman, WA, United States; ^3^ Terres Inovia–Institut technique des oléagineux, des protéagineux et du chanvre, Paris, France; ^4^ Université de Toulouse, INRAE, INP-ENSAT Toulouse, UMR AGIR, Castanet-Tolosan, France

**Keywords:** planting date, glycine max, varietal phenotyping, photoperiod, temperature

## Abstract

Developing new cropping strategies (very early sowing, crop expansion at higher latitudes, double cropping) to improve soybean production in Europe under climate change needs a good prediction of phenology under different temperature and photoperiod conditions. For that purpose, a simple phenology algorithm (SPA) was developed and parameterized for 10 contrasting soybean cultivars (maturity group 000 to II). Two experiments were carried out at INRA Toulouse (France) for parameterization: 1) Phenological monitoring of plants in pots on an outdoor platform with 6 planting dates. 2) Response of seed germination to temperature in controlled conditions. Multi-location field trials including 5 sites, 4 years, 2 sowing dates, and 10 cultivars were used to evaluate the SPA phenology predictions. Mean cardinal temperatures (minimum, optimum, and maximum) for germination were ca. 2, 30, and 40°C, respectively with significant differences among cultivars. The photoperiod sensitivity coefficient varied among cultivars when fixing Popt and Pcrt, optimal and critical photoperiods respectively, by maturity group. The parameterized algorithm showed an RMSE of less than 6 days for the prediction of crop cycle duration (i.e. cotyledons stage to physiological maturity) in the field trials including 75 data points. Flowering (R1 stage), and beginning of grain filling (R5 stage) dates were satisfactorily predicted with RMSEs of 8.2 and 9.4 days respectively. Because SPA can be also parameterized using data from field experiments, it can be useful as a plant selection tool across environments. The algorithm can be readily applied to species other than soybean, and its incorporation into cropping systems models would enhance the assessment of the performance of crop cultivars under climate change scenarios.

## Introduction

EU-28 needs to import about 40 million tons of soybean (seed equivalent) for feeding livestock, and humans to a lesser extent. In recent years, soybean production in Europe has largely increased reaching 2.7 million tons in 2018 (USDA, 2019), but remains insufficient for the increasing demand of conventional and organic grains and soyfoods. In the context of climate change, southern areas in Europe are expected to experience more frequent and severe drought and heat waves while northern areas may benefit from higher temperatures in late season (IPCC, 2014). Three cropping strategies could be proposed to grow soybean under these conditions: (i) early sowing to use less irrigation water in summer as escaping strategy (Maury et al., 2015); (ii) northward cultivation of the crop to extend its cropping area toward regions becoming more suitable with increasing temperatures (Olesen et al., 2011); or (iii) double cropping after cereals under late sowing to fully use the thermal time window (Seifert and Lobell, 2015). These new crop management systems using either early or late sowing and new cultivation areas will expose the crop to a wider range of thermal and photoperiodic conditions under higher latitudes (from 43° to 52° N).

Soybean is a thermophilic plant whose development rate is governed by three cardinal temperatures: minimum temperature (T0, also referred to as base temperature), below which soybean does not develop; optimum temperature (Topt), at which soybean develops at the highest rate; and maximum temperature (Tmax), above which development is stopped. It is difficult to determine a definitive T0 temperature from the literature. Reported T0 values differ greatly, ranging from 2.5 to 13.2°C (Grimm et al., 1993; Sinclair et al., 2005; Salem et al., 2007; Setiyono et al., 2007) with a potential variation of T0 during the cycle (Setiyono et al., 2007). These variations are explained by the diversity of studied cultivars and methods used. Optimum temperature has been reported between 22 and 32°C depending on the studies (Grimm et al., 1993; Boote et al., 1998a; Setiyono et al., 2007; Boote, 2011; Parent and Tardieu, 2012) while maximum temperature has been less explored, with reported values between 40 and 47°C (Salem et al., 2007; Setiyono et al., 2007).

The change in sowing dates, mostly tested on late maturity groups from III to VII (Bowers, 1995; Hu and Wiatrak, 2012; Salmeron et al., 2014), can place the early maturing soybean cultivars of interest in this study under suboptimal temperature conditions. Indeed, early planting increases the likelihood of exposing soybean to temperatures lower than T0 during early developmental stages (Meyer and Badaruddin, 2001; Maury et al., 2015), whereas late sowings for double cropping will lead to exposure to suboptimal temperatures for development at the end of the cycle and a risk of frost before harvest (Mahieu and Brinkman, 1990; Shapiro et al., 1992). The accumulation of thermal time for development can be calculated in different ways: (i) a linear equation, where daily thermal time is calculated by subtracting T0 from daily average temperature and then accumulated over the period of interest; (ii) a bilinear equation, which takes into account both T0 and Topt to obtain an upper bound temperature above which thermal time is not accumulated (Brisson et al., 2003); another bilinear triangular equation adds Tmax to impact the temperature accumulation between Topt and Tmax (Jones et al., 2003; Keating et al., 2003), and finally (iii) a nonlinear equation that uses the three cardinal temperatures and more closely represents temperature effect on biological functioning (Wang and Engel, 1998).

The characterization of cardinal temperatures is a basic step to parameterize thermal time equations, and the use of fast and reproducible methods for their determination is desirable. Parent et al. (2010) have shown that all the development processes respond in the same way to temperature. Therefore, it is convenient to choose a simple process for subsequent application to the whole plant. Salem et al. (2007) have chosen to characterize cardinal temperatures using pollen germination percentage and tube length, but it is also possible to use the germination process (Andreucci et al., 2016), which has the advantage of requiring only an incubator.

Soybean development rate is also strongly impacted by photoperiod due to its short-day plant behavior. Photoperiod regulates the onset of flowering (Garner and Allard, 1920; Hadley et al., 1984; Caffaro et al., 1988) and the duration of the phenological phases (Raper and Thomas, 1978; Kantolic and Slafer, 2001; Nico et al., 2015). An increase in day length above a given threshold, mainly related to the maturity group (Mcblain and Bernard, 1987; Xia et al., 2012; Lu et al., 2017), will slow down the development process. This characteristic has a particular impact on the post-flowering phase, which is more sensitive to photoperiod in late cultivars than in early ones (Major et al., 1975; Kantolic and Slafer, 2001). The type of growth (determinate vs. indeterminate) also results in different responses to photoperiod, especially between the onset of flowering (R1) and beginning seed fill (R5) stages where the final number of nodes is fixed for indeterminate cultivars. For these cultivars, long photoperiods will increase the length of R1 to R5 phase (Piper et al., 1996; Han et al., 2006; Kantolic et al., 2013).

Due to this response to photoperiod, a change in sowing dates will lead to development differences in soybean. Earlier planting will tend to initiate an early flowering, at a less advanced vegetative stage, since day length will be short enough to trigger it. The R1–R5 phase, which takes place when the day lengths are the longest, will be lengthened, allowing soybean to produce more pods and seeds (Kantolic and Slafer, 2001; Kantolic and Slafer, 2007; Kantolic et al., 2013; Nico et al., 2015). On the other hand, a late planting date will have the opposite effect, often with limitations of yield potential compared to conventional planting dates (Zhang et al., 2010; Hu, 2013; Clovis et al., 2015). Growth type, determinate or indeterminate, also plays a significant role. An indeterminate cultivar will have to be sown sooner, since the final number of nodes is reached at R5 (Piper et al., 1996).

In this study, we used a simple phenology algorithm accounting for temperature and photoperiod along with a protocol to determine the necessary parameters from controlled or field experiments. Models with different requirements of parameterization have been proposed that consider: (i) the total cycle (Piper et al., 1996), (ii) two phases—before and after a given stage, either R1 (Keating et al., 2003; Sinclair et al., 2005) or R5 (Brisson et al., 2003), (iii) a finer differentiation within phases (Setiyono et al., 2007; Salmerón and Purcell, 2016). Phenotyping these parameters is tedious, and mainly involves experimentation in pots inside culture chambers or greenhouses (Cregan and Hartwig, 1984; Ellis et al., 1992; Wang et al., 1998; Cober et al., 2014) or by extension or shortening of the photoperiod in the field through lights or shading systems (Kantolic and Slafer, 2001; Kantolic et al., 2013; Xu et al., 2013; Nico et al., 2015). It is also possible to use the natural variation of day length by testing several planting dates.

Depending on their responses to temperature and photoperiod, soybean cultivars are classified into different maturity groups (MGs). Today, thirteen maturity groups are included in the international classification system for soybean (Poehlman, 1987), classified from “000” for the very early to “X” (Roman numeral) for very late ones. Commonly cropped cultivars in Europe belong to maturity groups from 000 to II (Kurasch et al., 2017) while cultivars in the USA range from 00 to IX (Scott and Aldrich, 1970; Zhang et al., 2007), in Brazil from V to IX (Alliprandini et al., 2009), and in Argentina from II to IX (Dardanelli et al., 2006). The distribution of MGs for these regions depends mainly on the latitude, i.e. early cultivars are usually sown in high latitudes in northern hemisphere. MGs range from 000 to IX in China but are more related to eco-regions based on climatic and geographical conditions, cropping systems and season sowing types than to latitude (Yuesheng et al., 2006). The main difficulty encountered for extending soybean cropping to northern Europe and/or for modifying the sowing period is relative to the short-day behavior of the plant. As long photoperiods are usual in Europe, it is important to understand the temperature-photoperiod interactions of current and future cultivars.

The aim of this study was three-fold: (i) Develop a simplified phenotyping method for phenology in (semi-)controlled conditions (EXP1, EXP2), (ii) parameterize a simple phenology algorithm (SPA) using EXP1 and EXP2, and (iii) evaluate SPA using field experiments (EXP3).

## Material and Methods

### Experimental Design and Measurements

#### Cultivars’ Characteristics

Ten soybean cultivars representing diverse maturity groups and growth “types” (more or less indeterminate) used in Western Europe cropping systems were selected for this study. CVs ‘Isidor,’ ‘Santana,’ ‘Blancas,’ and ‘Ecudor,’ respectively from maturity groups I, I/II, and II, are usually grown under irrigation in South-West of France, which is the main French soybean production area. CVs ‘Klaxon,’ ‘Sultana,’ ‘RGT_Shouna,’ ‘ES_Mentor,’ and ‘Sigalia’ are considered as “very early” cultivars belonging to maturity groups from 000 to 00. They are usually grown as rainfed crops in the North-East of France, and in the North of Europe (Netherlands, Germany, Poland). Despite their small number, these cultivars have been chosen in order to represent the widest range of phenological characteristics (MG, growth type) among elite cultivars cultivated in Europe (**Table 1**). For example, ‘ES_Pallador,’ which belongs to the same MG as ‘Isidor,’ has a totally different type of growth, being very indeterminate. CV ‘Blancas’ has variable classification depending on location, being classified as MG I/II in Italy and II in South-West of France.

**Table 1:** Characteristics of the 10 cultivars used in this study.

Cultivar	Breeder	Maturity group	Growth type	Leaf type
KLAXON	RAGT 2N	000(0)	indeterminate	pointed-oval
RGT_SHOUNA	RAGT 2N	000	semi-indeterminate	pointed-oval
SULTANA	RAGT 2N	000	indeterminate	pointed-oval
ES_MENTOR	EURALIS SEMENCES	00	semi-indeterminate	rounded-oval
SIGALIA	RAGT 2N	00	semi-indeterminate to indeterminate	rounded-oval
ES_PALLADOR	EURALIS SEMENCES	I	indeterminate	lanceolate
ISIDOR	EURALIS SEMENCES	I	semi-determinate	big round leaves
SANTANA	RAGT 2N	I/II	indeterminate	rounded-oval
BLANCAS	CAUSSADE SEMENCES	II	indeterminate	rounded-oval
ECUDOR	EURALIS SEMENCES	II	indeterminate	big round leaves

They are ordered by maturity group (MG), from the earliest (‘Klaxon’–MG 000) to the latest maturing one (‘Ecudor’–MG II). ‘Klaxon’ was registered as MG 000 but according to the breeder’s expertise it was classified in this study as MG 0000. The studied germplasm comes only from French breeders. At least two contrasted cultivars by MG in terms of growth type and leaf type were kept in order to maximize the difference regarding photoperiod-temperature interactions.

#### Germination Rate Experiment to Determine Cardinal Temperatures—EXP1

This experimentation was conducted in 2018. Seeds of the 10 cultivars were incubated under eleven temperatures (3, 6.5, 10, 15, 20, 25, 30, 35, 37.5, 40, and 43°C). To do this, hundred seeds of each cultivar were placed in four 90 mm diameter Petri dishes, resulting in a total of 25 seeds by plate. To maintain sufficient moisture inside the plate, the seeds were placed between two filter papers and kept moist with 8 ml of purified (osmosis) water. Petri dishes were arranged on four trays (one for each replicate) in the incubator or the cold chamber depending on the temperature tested. Temperature was monitored using a sensor (THERMOCHRON DS1922T, Embedded Data Systems). Germinated seeds were counted and removed two or three times a day. One seed was considered as germinated when it presented a radicle, outside of the tegument, of at least 3 mm long (McDonald et al., 1988). Data produced during EXP1 are presented in the first tab of **Data Sheet 1** (**Supplementary Material**). To determine the cardinal temperatures for each cultivar from EXP1, we followed the method presented by Tribouillois et al. (2016). This method consists of using the Beta function described by Yin et al. (1995), which is fitted to experimental data to obtain the three cardinal temperatures (Equation 1). The Beta function adjustment was performed by simultaneously optimizing the three function parameters (mu, alpha, beta) with the Excel solver tool. A constraint was imposed on the T0 parameter so that it must be greater than or equal to 0 and Tmax were fixed according observations. The method used was the non-linear GRG with the goal of minimizing the RMSE between the simulated and the observed germination rate values.

Equation 1:

$$\frac{1}{t\left(G\right)}=\exp\left(\mu \right)\times {\left(T-T0\right)}^{\alpha }\;\times {\left(Tmax-T\right)}^{\beta }$$

Where μ, α and β are the model parameters; 1/t(G), the germination rate; T0, T, and Tmax, the minimum, actual and maximum temperatures respectively.

#### Response of Phenology to Temperature and Photoperiod in an Outdoor Pot Platform—EXP2

The 10 cultivars were grown on an automated outdoor pot platform (Heliaphen) designed for high-throughput plant phenotyping as previously described in Gosseau et al. (2019). As this experiment was set up to understand and model the temperature by photoperiod interactions, six planting dates were used: 17^th^ March (D1), 6^th^ April (D2), 16^th^ May (D3), 26^th^ June (D4), 24^th^ July (D5), and 1^st^ September 2017 (D6). These dates were chosen to cover the whole soybean growing season and to exhibit contrasting photoperiods and temperatures. Earlier (D1) and late (D6) planting dates experienced damaging cold stress and were not included for further analysis. In southwestern France, field planting dates generally occur between April 10^th^ and May 20^th^, corresponding to the planting dates D2 and D3 of this experiment. D1 and D5 represent new management practices. D1 begins to be tested in areas in order to escape water deficit around flowering and seed filling time (Maury et al., 2015). D5 is typical of double cropping systems, where soybean is planted after an early harvested crop (winter barley, pea) (Quinsac et al., 2014).

Seeds were sown in a greenhouse at a mean temperature of 20°C with natural day length to standardize the initial growth of seedlings. The most vigorous seedlings were transplanted in pots and transferred to the platform. This was also a way to prevent emergence losses on the platform due to possible poor seed quality. Before transferring the seedlings, an acclimation regime at a temperature around 10°C during 7 days was applied for the two first planting dates to prevent a risk of thermal shock. Seedlings which reached VC stage (first unifoliate leaf) were transplanted in individual pots filled with 15 L of P.A.M.2 potting substrate (Proveen, distributed by Soprimex, Chateaurenard, Bouches-du-Rhône, France) and 125 g of extended released fertilizer (Osmocote Exact High K 5-6M, ICL Specialty Fertilizers, distributed by Agri Garonne, Castelginest, Haute-Garonne, France). For each cultivar and planting date, three pots of five plants were prepared. The experiment consisted of a split-plot design with three blocks. The main plot consisted of planting date treatments and subplots of cultivars. The pots were organized by planting date to avoid shading effects on the youngest plants (cf. experimental design in **Figure A—Supplementary Material**). The 10 cultivars were randomized within each treatment-block combination. Pots were well-watered using drips. Hourly temperatures were recorded by an automatic weather station located within 150 m from the platform.

Plant phenology was recorded two times a week using Fehr and Caviness (1977) scale for vegetative and reproductive stages (description in **Table A—Supplementary Material**). Records began around VC stage and ended at R7 stage, which is the beginning of maturity (Setiyono et al., 2007). In total, the phenology of 900 plants was recorded and the day of appearance of each stage was calculated using the five plants in a pot (mean, standard deviation). Climatic and phenological data produced in EXP2 are sumarized in the second and third tabs of **Data Sheet 1** (**Supplementary Material**).

#### Field Experiments—EXP3

Eight field experiments were conducted in 2013, 2014, 2017, and 2018 in different experimental sites in the Toulouse region (43°N 1°E), namely Mondonville (2013, 2014), Rivières (2013, 2014), En Crambade (2013, 2014), and Auzeville (2017, 2018). The cultivars (CVs ‘Ecudor,’ ‘Isidor,’ ‘Santana’) were tested during the four seasons, but three more were added in 2017 (CVs ‘RGT_Shouna,’ ‘Sultana,’ ‘Blancas’), and three more in 2018 (CVs ‘Sigalia,’ ‘ES_Mentor,’ ‘ES_Pallador’). Two sowing dates (early and early + 1 to 1,5 month) were tested, except in 2018 where normal and late sowings were tested because of the wet spring. All the experiments were optimally irrigated using a decision tool (Terres Inovia, 2019), resulting in water applications from 65 to 161 mm. Daily temperatures were recorded by an automatic weather station located on each experimental site. At least four phenological stages were recorded when 50 percent of the plants in one plot had reached the stage: VE (emergence), R1 (first flower), R5 (beginning seed filling), R7 (beginning maturity). This information is summarized in **Table 2**. Climatic and agronomic data produced in EXP3 are sumarized in the last two tabs of **Data Sheet 1** (**Supplementary Material**).

**Table 2:** Summary of field experiments used for the validation of the simple phenology algorithm.

Year	Site	Planting type	Irrigation (mm)	Cultivars	Planting date	VE date	R1 date	R5 date	R7 date	Harvest date
2013	En Crambade	Conventional	145	Ecudor, Isidor, Santana	25-Apr	10-May	9-Jul	6-Aug	21-Sep	7-Oct
2013	En Crambade	Very Early	143	Ecudor, Isidor, Santana	15-Mar	13-Apr	29-Jun	23-Jul	12-Sep	7-Oct
2013	Mondonville	Conventional	86	Ecudor, Isidor, Santana	27-May	13-Jun	22-Jul	21-Aug	27-Sep	17-Oct
2013	Mondonville	Very Early	65	Ecudor, Isidor, Santana	22-Mar	16-Apr	17-Jun	12-Aug	15-Sep	14-Oct
2013	Rivieres	Conventional	125	Ecudor, Isidor, Santana	6-May	29-May	9-Jul	15-Aug	27-Sep	19-Oct
2013	Rivieres	Very Early	161	Ecudor, Isidor, Santana	22-Mar	17-Apr	15-Jun	5-Aug	8-Sep	2-Oct
2014	En Crambade	Conventional	120	Ecudor, Isidor, Santana	30-Apr	11-May	27-Jun	29-Jul	10-Sep	22-Sep
2014	En Crambade	Very Early	129	Ecudor, Isidor, Santana	14-Mar	7-Apr	11-Jun	14-Jul	30-Aug	13-Sep
2014	Mondonville	Conventional	0	Ecudor, Isidor, Santana	6-May	23-May	29-Jun	3-Aug	6-Sep	6-Oct
2014	Rivieres	Conventional	140	Ecudor, Isidor, Santana	6-May	16-May	1-Jul	1-Aug	16-Sep	9-Oct
2014	Rivieres	Very Early	100	Ecudor, Isidor, Santana	18-Mar	9-Apr	13-Jun	9-Jul	30-Aug	26-Sep
2017	Auzeville	Conventional	112	Ecudor, Isidor, Santana, Blancas, RGT_Shouna, Sultana	10-May	17-May	21-Jun	29-Jul	1-Sep	15-Sep
2017	Auzeville	Very Early	76	Ecudor, Isidor, Santana, Blancas, RGT_Shouna, Sultana	21-Mar	7-Apr	2-Jun	4-Jul	13-Aug	5-Sep
2018	Auzeville	Conventional	76	Ecudor, Isidor, Santana, Blancas, RGT_Shouna, Sultana, ES_Pallador, Sigalia, ES_Mentor	24-Apr	10-May	22-Jun	24-Jul	26-Aug	10-Sep
2018	Auzeville	Late	111	Ecudor, Isidor, Santana, Blancas, RGT_Shouna, Sultana, ES_Pallador, Sigalia, ES_Mentor	4-Jun	11-Jun	19-Jul	13-Aug	10-Sep	21-Sep

The four sites (En Crambade, Mondonville, Rivières, Auzeville) were located in the South-West of France, around Toulouse. All experiments were irrigated and had two planting dates, except Mondonville in 2014. The same three cultivars (‘Ecudor,’ ‘Santana,’ ‘Isidor’) were tested in 2013, 2014, 2017, and 2018. Respectively three and six cultivars were added in 2017 and 2018. Mean phenological stages needed for the study are reported for each experiment.

### Simple Phenology Algorithm

#### Description

A simple phenology algorithm (SPA) was developed to predict the occurrence of soybean development stages, accounting for diverse photoperiod and temperature conditions based on concepts introduced by Robertson (1968), and expanded by Wang and Engel (1998). Under non-limiting conditions of photoperiod and temperature, the optimal physiological development days (PDDopt_c,p_) to complete a given phenological phase and the number of calendar days are the same (Wang and Engel, 1998). When the conditions are limiting, the actual physiological development day (PDD) accumulated each day is less than 1, and the number of calendar days to complete the phase is larger. The accumulation of PDD required to complete a phenological phase for each cultivar (PDD_c,p_) is calculated using equation 2 (detailed in **Supplementary Material**). PDD_c,p_ was calculated for four development phases: VC–R7, VC–R1, R1–R5, and R5–R7.

Equation 2:

$$PDD_{c,p}=\;PDDopt_{\;c,p}\;/\sum_{i=1}^{PDD_{c,p}}(f\left(Ti\right)\times \;f\left(Pi\right)\;/\;PDD_{c,p})$$

Where:PDD_c,p_: Physiological Development Days of cultivar c and phase p (in calendar day) f(T) and f(P) are the temperature and photoperiod functions, respectively.

c: cultivar

p: phase

i: day number

#### Temperature Function

The temperature function (Equation 3) was described by Wang and Engel (1998).

Equation 3:

f(T)= 2(T−T0)α (Topt−T0)α−(T−T0)2α(Topt−T0)2α if T0≤T≤Tmaxf(T)=0if T<T0 or T>Tmax

Where:

T: average temperature of the day (°C).

T0, Topt, Tmax: cultivar-specific cardinal temperatures (°C).

The parameter α is calculated using Equation 4:

Equation 4:

α=log(2)/log[(Tmax−T0)/(Topt−T0)]

#### Photoperiod Function

The photoperiod function f(P) was calculated as shown in Equation 5.

Equation 5:

$$\begin{array}{ll} f\left(P\right)=1-{\left[\left(P-Popt\right)/\left(Pcrt-Popt\right)\right]}^{S} & if\;Popt\leq P\leq Pcrt\\ f\left(P\right)=1 & if\;Popt<P\\ f\left(P\right)=0 & if\;Popt>P \end{array}$$

Where Pcrt is the day length above which development rate is zero, Popt is the day length below which development rate is not limited, P is the day length including civil twilight, all in hours, and S is the sensitivity coefficient of the cultivar to photoperiod (S=0 for highest sensitivity; >100 for no sensitivity).

#### Parameterization

Plant parameters needed in SPA are presented in **Table 3**, obtained from three methods of acquisition: experimental data, literature, and optimization.

**Table 3:** Parameters used in Simple Phenology Algorithm (SPA).

Name	Unit	Abbreviation	Origin of data
Minimum temperature of development	(°C)	T0	EXP1
Optimal temperature of development	(°C)	Topt	EXP1
Maximum temperature of development	(°C)	Tmax	EXP1
Optimal daylength for maximum development	(h)	Popt	Literature
Critical daylength for zero development	(h)	Pcrt	Literature
Physiological development days in optimum conditions of temperature and photoperiod	d	PDDopt_c,p_	Optimization
Sensitivity coefficient to the photoperiod	unitless	S	Optimization

Plant parameters are divided into three types depending on their method of acquisition. Cardinal temperatures (T0, Topt, Tmax) were directly produced in EXP1 at a cultivar level. Popt and Pcrt have been adopted from (Setiyono et al., 2007), based on the cultivar’s MG. PDDopt_c,p_ and sensitivity coefficient to the photoperiod were optimized during the parameterization phase.

Once all other parameters were determined (i.e. cardinal temperatures, Popt and Pcrt—**Table 3**), PDDopt_c,p_ and S were set by simultaneous optimization over the entire life cycle VC–R7. The optimization was performed with the Excel solver using the non-linear GRG method (Newton Global). It was carried out in two steps: 1) simultaneous optimization of the minimum number of days for the VC–R7 phase (PDDopt_c,p_) and of the sensitivity to the photoperiod (S); 2) optimization of the minimum number of days for the R1–R5 and R5–R7 phases while maintaining the value of S as determined in (1). The objective of the optimization was to minimize the RMSE between predicted and actual day of appearance of R7 for all planting dates of a given cultivar.

#### Evaluation of SPA

SPA was evaluated based on a set of field data corresponding to 75 cases including 5 sites, 4 years, 2 sowing dates, and 10 cultivars (cf **Table 3**). The parameterized SPA was used to predict the phenology in these independent conditions. As we did not have a VC rating in 2013 and 2014, we estimated it by considering that it was reached at 50°C.day^-1^ after VE (as observed in 2017 and 2018). Indeed, only thermal effects play a role at this stage of development (Hodges and French, 1985).

#### Statistical Analysis

The effects of cultivars, planting dates, and their interactions were identified by analysis of variance using a two-way ANOVA performed with R software (version 3.2.2; [R Core Team {2015} R: a language and environment for statistical computing. R Foundation for Statistical Computing, Vienna]). Comparison of means was done with post-hoc Student-Newman-Keuls (SNK) tests with a significance level of p < 0.05.

#### Indicators of Algorithm Performance

The performance of SPA was evaluated with several indicators including RMSE (Root Mean Square Error), MBE (Mean Bias Error), RRMSE (Relative Root Mean Square Error), and EF (Efficiency). Equations of performance indicators are presented in **Table 4**.

**Table 4:** Performance indicators used for the evaluation of SPA. Equations and units are reported.

Performance Indicator	Equation	Unit
Root Mean Square Error (RMSE)	$RMSE\;=\;\sqrt{\frac{1}{n}\sum_{i=1}^{n}({\hat{y}}_{i}-y_{i}{)}^{2}}$	days
Mean Bias Error (MBE)	$MBE=\frac{\sum_{i=1}^{n}({\hat{y}}_{i}-y_{i})}{n}$	days
Relative RMSE (RRMSE)	$RRMSE=RMSE/\bar{y}$	unitless
Efficiency (EF)	$\left[EF=1-\frac{\sum {\left(y_{i}-{\hat{y}}_{i}\right)}^{2}}{\sum {\left(y_{i}-{\bar{y}}_{i}\right)}^{2}}\right]\times 100$	%

Where *y_1_* is the observed value for the i observation, ${\hat{y}}_{i}$ is the predicted value, and $\bar{y}\;$ the average of observations.

## Results

### Cardinal Temperatures of Germination

**Figure 1:**
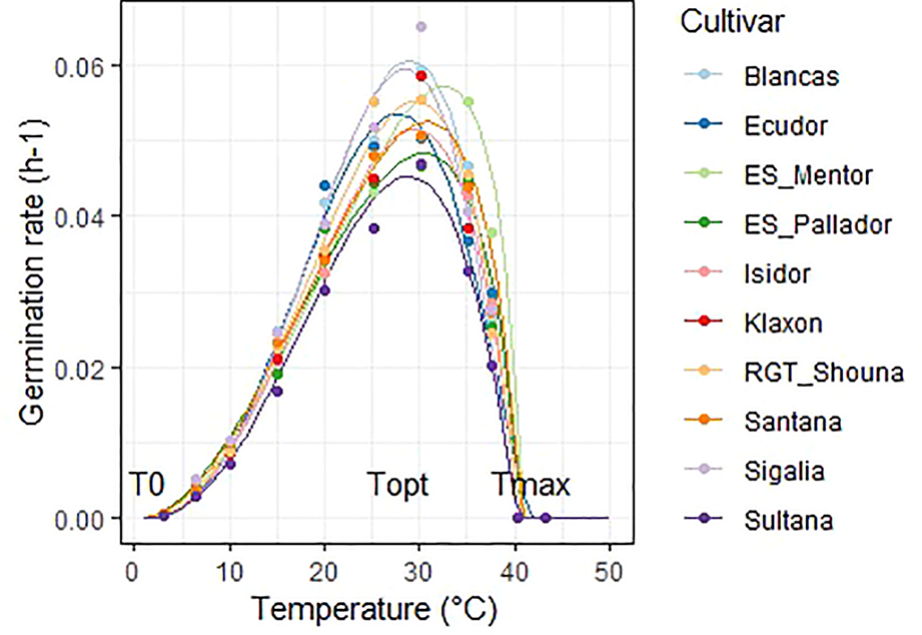
Graphical display of the Yin adjustments for all cultivars at 50% of germination. Dots represent mean observed data and lines the adjusted Yin function. Position of cardinal temperatures (T0, Topt, Tmax) are illustrated on the graph.

The Yin model fitted well the observation data; these are are available in **Data Sheet 1 (Supplementary Material)**. Determination coefficients R² ranked from 0.95 to 1. The germination rate progressively increased from the minimum temperature to the optimum temperature and then decreased. The maximum germination rates varied according to the cultivars (**Figure 1**).

The average T0, Topt, Tmax of all the 10 cultivars were 2, 28.1, 40.6°C respectively. The lowest temperature tested was 3°C and having no point of reference we chose to set T0 to 2 °C. The final germination percentage was not significantly affected in the 6.5 to 40 °C temperature range under these experimental conditions (**Table B—Supplementary Material**).

Cultivar differences were significant for optimum and maximum temperatures (**Table 5**). The optimum temperature was the most discriminating, and allowed to separate four significantly different groups at the p < 0.05 threshold. ‘ES_Mentor’ is the cultivar which takes the best advantage of high temperatures (Topt = 32.1°C) while ‘Ecudor’ requires a lower temperature (28.1°C). In contrast to Topt, Tmax for ‘Ecudor’ was the highest of the cultivar group tested (42°C), with a majority of cultivars being between 40.3 and 41°C.

**Table 5:** Estimates (and standard errors [±]) of optimum (Topt) and maximum (Tmax) temperatures obtained by the Yin function for the germination of the 10 soybean cultivars studied.

MG	Cultivars	Topt (°C)			Tmax (°C)		
000(0)	Klaxon		29.8 ± 0.11	c		40.4 ± 0.31	c
000	RGT_Shouna		29.8 ± 0.23	c		40.7 ± 0.38	bc
000	Sultana		29.6 ± 0.48	c		40.4 ± 0.31	c
00	ES_Mentor		32.1 ± 0.76	a		40.4 ± 0.31	c
00	Sigalia		29.2 ± 0.50	c		40.4 ± 0.31	c
I	ES_Pallador		30.7 ± 0.21	b		40.3 ± 0.00	c
I	Isidor		29.8 ± 0.46	c		40.3 ± 0.00	c
I/II	Santana		30.5 ± 0.40	b		40.3 ± 0.00	c
II	Blancas		29.7 ± 0.54	c		41.0 ± 0.00	b
II	Ecudor		28.1 ± 0.64	d		42.0 ± 0.00	a
Average			29.9 ± 0.43			40.6 ± 0.16	

T0 was set to 2°C. In a column, means followed by common letter(s) are not significantly different at p < 0.05 level by Student-Newman-Keuls test.

### Temperature and Photoperiod Effects on Plant Phenology in the Outdoor Platform

#### Temperature and Photoperiod Conditions

The different planting dates did not experience the same photoperiod and temperature conditions. For example, D1, D2, and D3 met a varying photoperiod, increasing at the beginning and decreasing at the end of the cycle. D4, D5, and D6 only met a decreasing photoperiod. The temperature and photoperiod conditions encountered during EXP2 are presented in **Figure B (Supplementary Material)**.

#### Impact of Planting Date and Cultivars on the Duration of Phenological Phases

The variance analysis of cycle duration (from VC to R7 stage) in calendar days (CD VC–R7) showed a highly significant effect (p < 0.001) of planting date, cultivar, and the interaction of these two factors (**Table 6**). Block effect and Block X Cultivar interaction were not significant. Total cycle duration showed an amplitude from 100 to 155 days across planting dates, all cultivars combined. Results are available in **Data Sheet 1 (Supplementary Material)**.

**Table 6:** Analysis of variance of cycle duration from VC to R7 stage in calendar days (CD VC–R7) of 10 cultivars of soybean for five planting dates in outdoor pot experiment (EXP2).

	CD VC–R7	
Source of variation	Df	MS
Planting date	4	5118***
Block	2	48.
Cultivar	9	900***
Planting date × Cultivar	36	59***
Planting date × Block	8	58**
Block × Cultivar	18	20
Residuals	66	17

Planting dates analyzed were D1 to D5, as D6 did not reach R7 stage. (.), (**), and (***) significant at 0.1, 0.01, 0.001 levels, respectively.

Df: Degree of freedom; MS: Mean Square.

### Simple Phenology Algorithm

#### Parameterization of SPA

**Figure 2:**
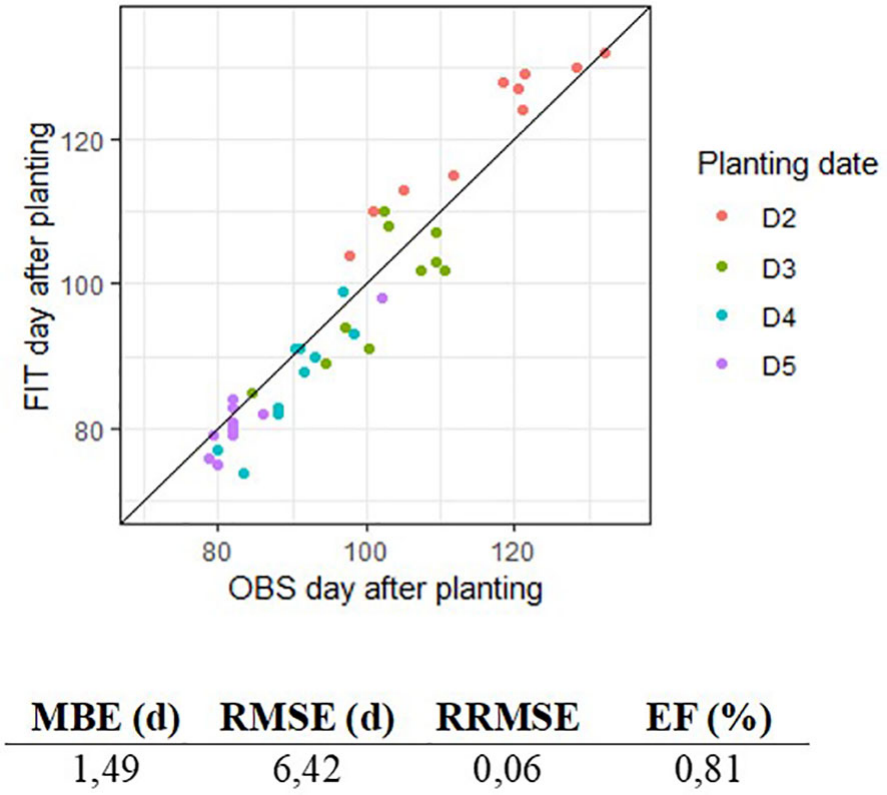
Comparison of fitted (FIT) and observed (OBS) day of appearance of R7 stage, for algorithm parameterization on outdoor pot experiment data (EXP2). Colors represent the four planting dates D2 to D5.

The parameterization led to satisfactory prediction of the dates of stages. **Figure 2** shows predicted and observed occurrence (day of year) of R7 stage for planting dates D2 to D5. Despite a slight overestimation for D2, predicted values were close to those observed, and the quality of adjustment was satisfactory with an efficiency of 81% and an RMSE of 6.42 days for the appearance of R7 stage since the day of planting. Parameter values are presented in **Tables 5**, **6**, **7**.

**Table 7:** Parameters used for each phase. The temperatures T0, Topt, and Tmax were those determined during EXP2 (**Table 5**).

Cultivar	GM	Pcrt (h)	Popt (h)	S	PDDopt _cp_ (d)			
					VC–R7	VC–R1	R1–R5	R5–R7
Klaxon	000(0)	20,5	13,0	1,29	41,9	11,4	12,7	19,2
RGT_Shouna	000	20,0	13,0	1,16	42,8	10,4	13,2	19,9
Sultana	000	20,0	13,0	1,18	41,9	10,5	11,9	20,4
ES_Mentor	00	19,50	12,50	1,47	31,3	7,1	9,8	15,3
Sigalia	00	19,50	12,50	1,06	42,8	10,0	11,6	21,9
ES_Pallador	I	19,00	12,50	1,00	32,7	8,3	11,3	13,6
Isidor	I	19,00	12,50	1,23	41,9	10,6	12,8	19,0
Santana	I/II	19,0	12,5	1,32	42,9	10,2	16,4	17,2
Blancas	II	18,3	12,0	1,50	42,1	10,3	15,7	16,7
Ecudor	II	18,3	12,0	1,35	46,3	10,1	16,6	18,9

Pcrt and Popt were calculated from the bibliography depending on the maturity group of the cultivars. S and PDDopt_c,p_ have been optimized for the VC–R7 phase. For the three intermediate phases (VC–R1, R1–R5, R5–R7), only PDDopt_c,p_ changed.

#### When Does Photoperiod and Temperature Affect Soybean Phenology?

**Figure 3:**
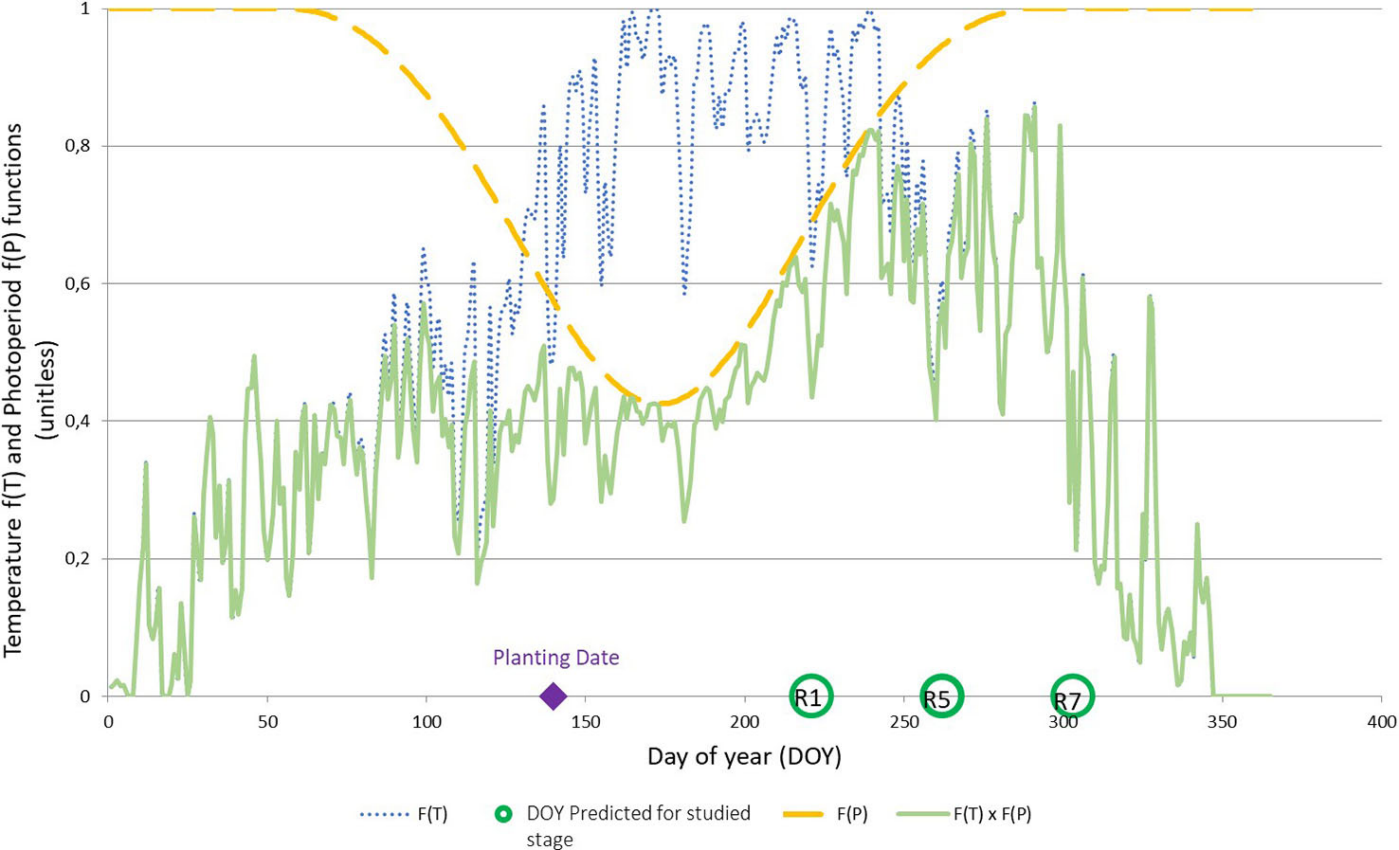
Graphical representation of the temperature function f(T), dotted blue line; photoperiod function f(P), dashed yellow line; and their multiplicative effect f(T) × f(P), solid green line, with parameterization parameters of ‘Ecudor’ cultivar for planting date D3 (10^th^ may). The planting date as well as the stages R1, R5, and R7 are reported on the abscissa axis.

The parameterization of SPA made it possible to highlight the phases impacted by limiting temperatures or photoperiods for each cultivar. As an example, **Figure 3** shows individually the effects of f(T) and f(P) and their multiplicative effects on the development of CV ‘Ecudor’ for planting date D3 on the outdoor platform. The photoperiod acts on development at a time when the temperature would not be limiting (f[T] ~ 1) around DOY 170, the value of f(P) being at its minimum at ~0.4. For earlier planting around DOY 80, soybean development would be more impacted by temperature with a value of f(T) close to 0.4 and a f(P) of 0.8.

**Figure 4:**
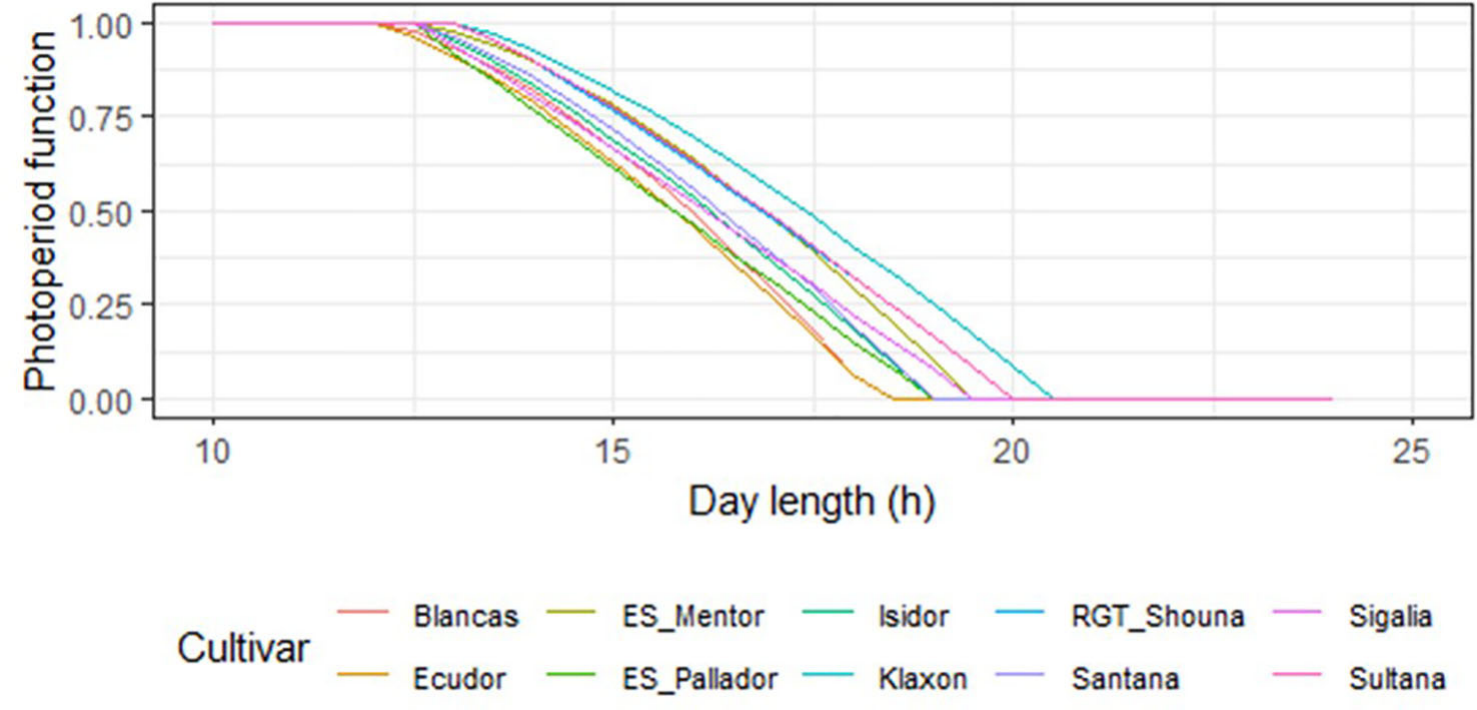
Photoperiod function representation for all cultivars using the calibration parameters. Maturity groups of the cultivars are the following: 000: ‘Klaxon,’ ‘Sultana,’ ‘RGT_Shouna’; 00: ‘Sigalia,’ ‘ES_Mentor’; I: ‘ES_Pallador,’ ‘Isidor’; I/II: ‘Santana’; II: ‘Blancas,’ ‘Ecudor’.

Sensitivity coefficient (S—**Table 7**) ranged from 1 for ‘ES_Pallador’ to 1.50 for ‘Blancas.’ However, this sensitivity was also impacted by Popt and Pcrt, which were determined according to the maturity group. **Figure 4** shows the photoperiod function for all cultivars with this calibration. Overall, there is a trend for the response to photoperiod to decrease from late to early cultivars.

#### Evaluation of SPA With Independent Field Data

**Figure 5:**
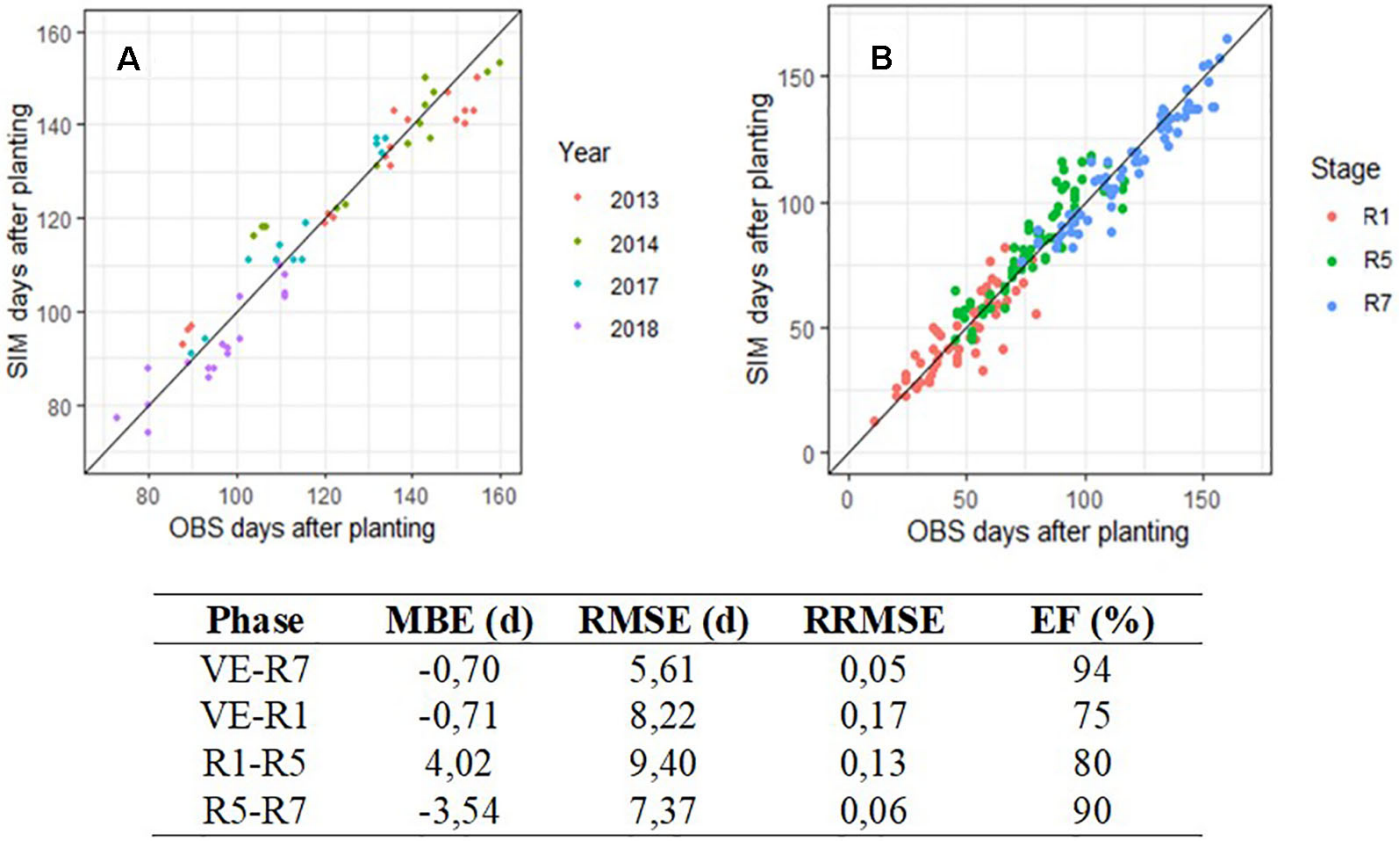
Observed (OBS) and predicted (SIM) days after planting for maturity (R7), all cultivars combined **(A)** and for three successive phenological phases **(B)**. In graph A, colors indicate years from 2013 to 2018 on data produced at INRA Auzeville, En Crambade, Rivières, and Mondonville. In graph B, colors indicate dates appearance of R1, R5, and R7 with simulations beginning respectively at VE, R1, and R5. Line 1:1 is reported on both graphs A and B. Table below shows the indicators of performance of SPA during its field evaluation.

**Figure 5A** shows the evaluation results for VE–R7 phase and all years combined. The algorithm predicted the occurrence of the R7 stage with 5.6 days of error on average and a prediction efficiency of 94%. The prediction was satisfactory, even for early planting resulting in a date of appearance of stage R7 outside the conventional range, here before calendar day 210. Data were a little more scattered during 2014 testing year. The same analysis was performed by phases, and the results are shown in **Figure 5B**. Predictions by phases gave variable levels of performance, but always lower than for the entire cycle. Performance indicators for each phase are reported in the table below the two graphs. VE–R1 phase tended to be underestimated for short phase length (before calendar day 160) while R1–R5 phase was the only stage overestimated by SPA, with a MBE of 4.02 days.

## Discussion

The aim of this study was to develop and apply a phenology phenotyping method coupled with a simple phenology algorithm for soybean cultivars. Phenotyping was divided into two complementary protocols to determine a) cardinal temperatures (EXP1) and b) development response to temperature and photoperiod (EXP2). Data produced in these experiments were used to parameterize SPA (Simple Phenology Algorithm), which was evaluated with independent data from field experiments.

### Is the Maturity Group Sufficient to Characterize the Phenology of a Cultivar?

The 10 cultivars tested showed differences in phenology in response to the planting dates tested in EXP2. To compare cultivar cycle lengths in a way familiar to breeders, we also calculated cumulative thermal times based on cardinal temperatures and f(T). The analysis of variance showed a significant effect of planting date, cultivars, and interaction of both on thermal time accumulation for p < 0.001. **Table 8** lists the cumulative thermal time to reach R7 for each planting date and cultivar. Differences in thermal time accumulation across cultivars (cultivar amplitude) decreased when delaying planting date; it reached its maximum at date D2 and its lowest at D5. The MG classification of cultivars in Europe is usually performed using D3 planting date (10^th^ may). As the cultivars are normally not tested for late planting (D5), which would be useful in double cropping decisions, breeders may choose to recommend an early and less productive cultivar while a later one could take advantage of the effect of the photoperiod to reach maturity. We showed in this study that with a planting date around 20^th^ July (D5), a cultivar from MG I could reach beginning maturity (R7) at the same cumulative thermal time than a cultivar from MG 000 (**Table 8**). This behavior was neither expected nor yet studied in our area. It would be interesting to characterize the cultivars not by their cycle length in days for a given planting date, but by their PDDopt_c,p_, which would allow breeders to have another criteria for selection. Subsequently, cultivar suitability maps could be developed and characterized, as described by Kurasch et al. (2017). In addition, photoperiod sensitivity of cultivars appears variable among maturity groups. Indeed, two cultivars of the same maturity group can be classified into two different groups according to the SNK test when considering the cumulative thermal time needed to complete the cycle.

**Table 8:** Thermal time accumulation from VC (unifoliate leaves) to R7 (beginning maturity stage), by cultivar and planting date.

Maturity group	Cultivar	D1	D2	D3	D4	D5	Mean	Planting date amplitude
000(0)	Klaxon	1691	1717	1881	1794	1513	1719 d	367
000	RGT_Shouna	1787	1891	2174	1845	1518	1843 cd	656
000	Sultana	1769	1818	2143	1735	1514	1796 d	630
00	ES_Mentor	1311	1412	1705	1280	1063	1354 e	642
00	Sigalia	2360	2544	2609	2088	1767	2274 a	842
I	ES_Pallador	1993	2007	2294	1729	1407	1886 c	886
I	Isidor	2265	2210	2414	1831	1537	2051 b	877
I/II	Santana	2134	2346	2296	1829	1598	2041 b	748
II	Blancas	2415	2670	2487	2193	1801	2313 a	869
II	Ecudor	2503	2591	2525	2153	1798	2314 a	793
**Mean**		2023 c	2121 b	2253 a	1848 d	1552 e			
**Cultivar amplitude**		1192	1258	904	913	737			

Values followed by common letter(s) are not significantly different at p < 0.001 level by SNK test. Planting date and genotypic amplitude are the calculation of maximum minus minimum values from each line and each column, respectively.

### Simple Phenotyping Method

EXP1 was used to determine the cardinal temperatures of cultivars. As presented earlier, fitting the non-linear function described by Yin et al. (1995) led to setting T0 = 0°C for all cultivars tested. However, by fitting a linear function on our data, we found T0 values between 4.5 and 6.1°C (data not shown), which agree with Covell et al. (1986), who also used a linear model. During EXP1 all cultivars germinated at 3°C, indicating that soybean can develop at lower temperature than reported T0 values based on linear fitting. It also illustrates that T0 is not truly a physiological parameter but rather influenced by the fitting method used. Taking into account the observed values, we therefore decided to set T0 at 2°C to get closer to biological reality. Topt, which exhibited a significant difference among cultivars, did not follow any MG-related order. A cultivar-dependent value of Topt has not been reported to our knowledge for soybean. According to Parent and Tardieu (2012), Topt would depend only on the cultivated species, these authors finding no differences within maize, wheat, and rice cultivars and lines. In order to confirm this finding, it would be interesting to study the response to temperature for a different process, for example the development phase VC–V3, as used by Parent and Tardieu (2012) in their study. The determined Tmax are in the range of what has already been published (Salem et al., 2007; Setiyono et al., 2007). We carried out a supplementary analysis with an nls model performed with R software, with or without genotypic effect in order to test the genotypic effect of cardinal temperatures. The values obtained according to Aikake’s information criterion were −3417 and −3323 respectively, indicating the interest in keeping the information at the cultivar level. In addition, genotypic differences for the different parameters were highly significant (p < 0.001, **Table 5**). The calculation of Topt and Tmax by germination percentage and their comparison by a *post-hoc* test gave the same rankings as the method initially used. The method we used (fitting a model per cultivar) has the advantage of determining the cardinal temperatures of a cultivar independently of the other cultivars, which can be useful for phenotyping a new cultivar. Using the cardinal temperatures determined during the germination experiment for the entire cycle was preferred for the sake of simplification (experimentation, modeling). However, it was theoretically justified by the work of Parent and Tardieu (2012); these authors showed that the cardinal temperatures remained essentially the same regardless of the development process (at least until flowering). By extension, we have applied it to the different phases of the cycle, including the post-flowering period. This assumption was not refuted when applying the model to the different phases then comparing the resulting simulations to field observations. However, other models (e.g. Setiyono et al., 2007) use different cardinal temperatures depending on the phases of the cycle. It may be possible to improve phase prediction by doing this with SPA but this would need specific experiments in controlled conditions or very wide experimental field network to determine them.

Using EXP2, we have phenotyped the response to photoperiod of contrasting cultivars grown in Europe for the developmental phase VC–R7. Phenotyping phenology in a pot experiment over the complete life cycle and including several planting dates is not common. In most cases, under these conditions, experiments are limited to a maximum of R1 stage (Wang et al., 1998; Sinclair et al., 2005). This experiment made it possible to position the soybean cycle over day length ranging from 9 h (D5) to 16.5 h (D2 and D3). Pot cultivation simplified stage ratings, which could have been laborious in the field. The tested density of 5 plants per pot of 28 cm diameter corresponds to a density of 83 plants.m^-2^ uniformly distributed on the platform. For comparison, in the field, the sowing rate is close to 50 plants.m^-2^ spaced 50 cm apart and therefore arranged tighter on the same row. We increased the density of plants in the pots mainly to increase the number of repetitions per cultivar while maintaining a density that does not have a known negative impact on the agronomic performance of the crop in the field (Edwards and Purcell, 2005). Certainly, EXP2 did not allow soybean to be exposed to continuous optimal photoperiod and temperature conditions throughout the cycle, which would have made it possible to directly determine PDDopt_c,p_ of each phase, therefore not requiring optimization. Experiments in a growth chamber would allow collecting this information, but this requires the necessary equipment and such a long cycle (VC–R7) has never been tested in this type of experiments to our knowledge.

### Simple Algorithm of Phenology

The parameterization of the algorithm gave satisfactory results, even if the early planting date D2 cycle length was overestimated by 5.5 days on average (data not shown). The performance during evaluation was satisfactory for VE–R7 phase, with an efficiency of 94% and a RMSE of 5.61 days. This compares well with the performance of the more detailed and harder to parameterize approach of Setiyono et al. (2007) (RMSE of 3 days) and soil-crop models like STICS and CropGro (RMSE ~10 days) (Boote et al., 1998b; Sojamip research project, http://www6.inra.fr/sojamip). These results show that it is possible to use a semi-controlled phenotyping method such as an automated outdoor platform to parameterize an algorithm that can be used to predict phenological stages in the field. This could be useful as a plant breeding tool. Because the algorithm has been developed based on a limited range of MGs, it is not possible to check its suitability for later MGs with our data. Nevertheless, maturity groups tested correspond to most MGs grown in Europe.

The duration of R1–R5 phase was overestimated for early plantings by 4 days on average. Longer photoperiods were met during this stage, the summer solstice taking place on DOY 172 with 16.5 h a day at a latitude of 43°N. This means that the photoperiod function f(P) applied had a strong impact on the development rate (f(P) values around 0.4) on this phase. In SPA, we chose to use a non-linear photoperiod function, unlike other models such as STICS (Brisson et al., 2003). The effect of f(P) in SPA is therefore greater for photoperiods close to Pcrt for long photoperiods compared to a linear formalism. Popt and Pcrt have been adopted from Setiyono et al. (2007), but it would be desirable to re-evaluate these thresholds with experiments such as the one presented by Daba et al. (2016). The optimization of the photoperiod sensitivity coefficient (S) made it possible to highlight differences in sensitivity within the same maturity group, which have the same Popt and Pcrt. This element must be taken into consideration by breeders when adapting cultivars to new management practices or new latitudes.

To test if the approach could be simplified by replacing cultivar-specific Topt by soybean-specific Topt, we calculated an average value of Topt across the 10 cultivars then re-estimated the values of PDDopt_c,p_, and S parameters. The prediction quality of VC–R7 duration was compared at field level (75 data points) for the two sets of parameters, either cultivar- or soybean-specific (**Table C—Supplementary Material**). RMSE (6.20 vs. 5.61 days) and MBE (−2.42 vs. −0.70) indicators performances were lessened when using soybean- instead of cultivar-specific Topt values respectively. Therefore using a cultivar-specific calibration in this study was justified. However, due to the slight decrease of the performance of SPA with average Topt, end-users could prefer adopt a soybean-specific Topt value for a direct and simpler application of the phenological algorithm.

One important goal of this study was to develop and evaluate a simple algorithm of phenology. Its simplicity comes from the low number (5) of parameters (photoperiodic thresholds and cardinal temperatures), observed or adopted from literature, plus two additional parameters obtained by optimization (PDDopt_c,p_ and S) based on observed phenology data. Other models can predict phenology well, but they are more difficult to parameterize (Setiyono et al., 2007) or they reach up to R1 only although with more detailed analysis (Sinclair et al., 2005).

## Conclusion

In this study we provide a path for soybean cultivar phenology × environment × crop management (sowing date) advice by: (1) developing a simple algorithm of phenology; and (2) proposing a simple method in semi-controlled conditions for phenotyping cultivar phenology and determining parameters for the algorithm. SPA allows users to quantify the effects of temperature and photoperiod on soybean phenology that affect the possible extension of the crop to northern Europe. It will also help to define new cultivation areas and management practices (i.e. early or late sowing) according to cultivar characteristics in the context of climate change. Because SPA is based on observations, it is possible to parameterize SPA directly from phenology data produced in field experiments, enhancing the value of these experiments and assisting plant selection efforts. For the same reason, SPA can be parameterized for other crop species without modification. Finally, integration of SPA into cropping system models can enhance the performance of these models for assessing the adaptation of cultivars to climate change scenarios and changing management options.

## Data Availability Statement

All datasets generated for this study are included in the article/**Supplementary Material**. 

## Author Contributions

CS, COS, LC, PD, and PM, designed the research. PD, LC, and PM acquired the funding for the research project. CS and CC performed the experiments. COS designed the algorithm. CS, COS, PD, and PM analyzed the data. CS, COS, PD, and PM wrote the manuscript.

## Funding

The authors thank the Occitanie / Pyrénées-Méditerranée region and Terres Inovia for financial support of this research.

## Conflict of Interest

The authors declare that the research was conducted in the absence of any commercial or financial relationships that could be construed as a potential conflict of interest.
