# Supplementary material for: Combining Simple Phenotyping and Photothermal Algorithm for the Prediction of Soybean Phenology: Application to a Range of Common Cultivars Grown in Europe
Source: Front Plant Sci. 2020 Jan 29;10:1755. doi: 10.3389/fpls.2019.01755 (PMC7000526; doi:10.3389/fpls.2019.01755)
Supplement: Supplementary file 2 [file DataSheet_2.docx]

Supplementary Material

Schoving et al., 2020. Combining simple phenotyping and photothermal algorithm for the prediction of soybean phenology: application to a range of common cultivars grown in Europe.

# Supplementary Data

*Detailed calculation of the Physiological Development Days*

Calculation of PDD_c,p_ (in calendar day) which is the Physiological Development Days (PDD) required to complete the phenological phase “p” for the cultivar “c”. Below are the detailed equations for the calculation of PDD_c,p_ on the first day of the phenological phase (d=1), on day “d” of the phenological phase (1 ≤ d ≤ PDD_c,p_ ) , and on day d = PDD_c,p_ (the phenological phase is then performed, see equation 1 in the manuscript).

On day d=1:

$${PDD}_{c,p}= {PDDopt}_{c,p} / (f\left( T1 \right)x f\left( P1 \right) )$$

On day d (1 ≤ d ≤ PDDc,p):

$${PDD}_{c,p}= {PDDopt}_{c,p} / \sum_{i=1}^{d} (f\left( Ti \right)x f\left( Pi \right)/ d )$$

On day d=${PDD}_{c,p}$ (referenced as equation 1 in the manuscript):

$${PDD}_{c,p}= {PDDopt}_{c,p} / \sum_{i=1}^{{PDD}_{c,p}} (f\left( Ti \right)x f\left( Pi \right)/ {PDD}_{c,p} )$$

On day d=${PDD}_{c,p}$ , the equation can be written in simplified form:

$$PDDopt = \sum_{i=1}^{{PDD}_{c,p}} (f\left( Ti \right)x f\left( Pi \right) )$$

Where :

PDD_c,p_ : Physiological Development Days (PDD) required to complete the phenological phase “p” for the cultivar “c” (in calendar day)

PDDopt_c,p_ : Physiological Development Days (PDD) required to complete the phenological phase “p” for the cultivar “c” in optimal conditions (in calendar day)

f(T) and f(P) are the temperature and photoperiod functions, respectively (in fraction of calendar day)

c : cultivar; p : phase; i: day number

# Supplementary Figures and Tables

## Supplementary Figures


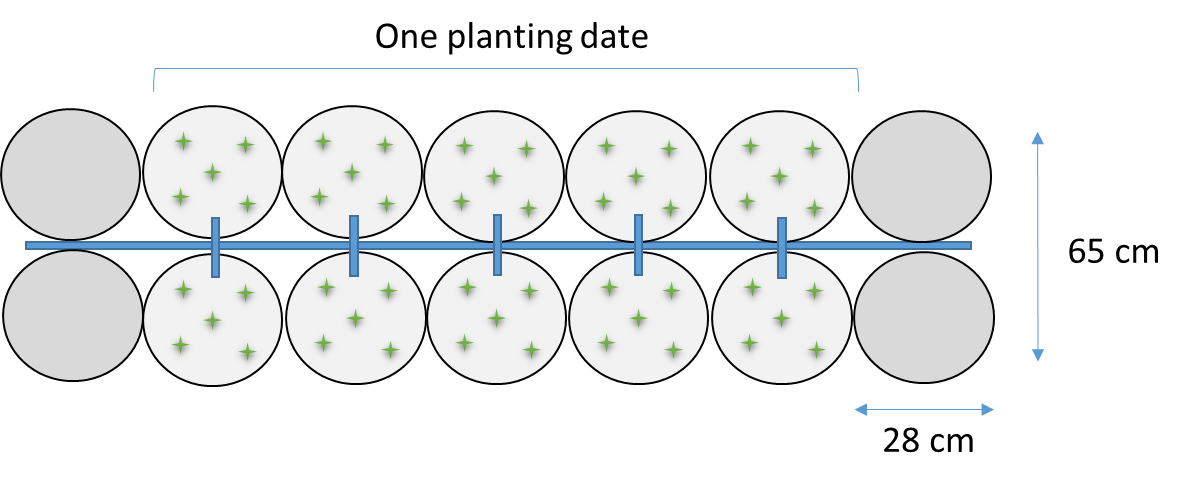


**Figure A**: Experimental design of EXP2. Each planting date was separated by the others by an empty pot (in grey). Five plants were planted by pot, resulting in a density of 83 plants by square meter.


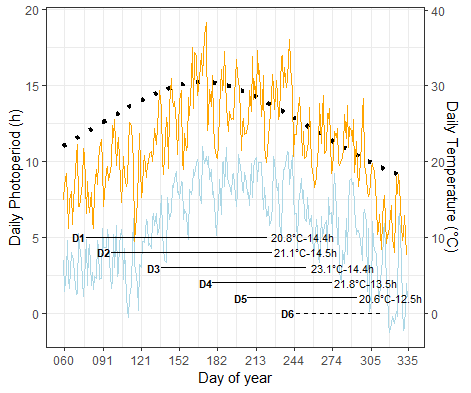


**Figure B**: Daily photoperiod and temperature during outdoor pot experiment from calendar day 59 (2017-02-28) to 334 (2017-11-30). Dotted bold line: daily photoperiod; Blue line: daily minimum temperature; Orange line: daily maximum temperature. The cycle lengths of the 6 planting dates are represented from VC (unifoliate leaves) to R7 (beginning maturity), except for D6 which ended around R4 stage. Average temperature and daylength during the cycle VC-R7 are reported at the end of each line.

## Supplementary Tables

**Table A**: Soybean development stages used in this study. Adapted from Fehr and Caviness (1977).

| **Vegetative stages** | **Abbreviated stage title** | **Description** |
| --- | --- | --- |
| VE | Emergence | Cotyledons above the soil surface |
| VC | Cotyledon | Unifoliate leaves unrolled sufficiently so the leaf edges are not touching |
| **Reproductive stages** |  |  |
| R1 | Beginning bloom | One open flower at any node on the main stem |
| R5 | Beginning seed | Seed 3mm (1/8 inch) long in a pod of the four uppermost nodes on the main stem with a fully developed leaf |
| R7 | Beginning maturity | One normal pod on the main stem that has reached its mature pod color |

**Table B**: Final germination percentages for all temperatures tested in EXP1.

| **Cultivar/Temperature (°C)** | **3** | **6.5** | **10** | **15** | **20** | **25** | **30** | **35** | **37.5** | **40** | **43** |
| --- | --- | --- | --- | --- | --- | --- | --- | --- | --- | --- | --- |
| Klaxon | 100 | 100 | 100 | 100 | 100 | 100 | 100 | 100 | 100 | 100 | 20 |
| RGT_Shouna | 100 | 100 | 100 | 100 | 100 | 100 | 100 | 100 | 100 | 100 | 50 |
| Sultana | 100 | 100 | 100 | 100 | 100 | 100 | 100 | 100 | 100 | 100 | 10 |
| ES_Mentor | 75 | 100 | 100 | 100 | 100 | 100 | 100 | 100 | 100 | 100 | 20 |
| Sigalia | 100 | 100 | 100 | 100 | 100 | 100 | 100 | 100 | 100 | 100 | 19 |
| ES_Pallador | 100 | 100 | 100 | 100 | 100 | 100 | 100 | 100 | 100 | 100 | 3 |
| Isidor | 100 | 100 | 100 | 100 | 100 | 100 | 100 | 100 | 100 | 100 | 0 |
| Santana | 100 | 100 | 100 | 100 | 100 | 100 | 100 | 100 | 100 | 100 | 3 |
| Blancas | 100 | 100 | 100 | 100 | 100 | 100 | 100 | 100 | 100 | 100 | 34 |
| Ecudor | 100 | 100 | 100 | 100 | 100 | 100 | 100 | 100 | 100 | 100 | 18 |

**Table C**: Performance of SPA depending on Topt parameter values: cultivar-specific vs soybean-specific (averaged across the 10 cultivars). The prediction quality of the VC-R7 phase duration in EXP3 (field) was tested with a re-estimation of PDDopt_c,p_, and S parameters with soybean-specific Topt.

| **Type** | **MBE (d)** | **RMSE (d)** | **RRMSE** | **EF (%)** |
| --- | --- | --- | --- | --- |
| Cultivar-specific | -0,70 | 5,61 | 0,05 | 94 |
| Soybean-specific | -2.42 | 6.20 | 0,05 | 93 |
